# Experimental study of the remediation of acid mine drainage by Maifan stones combined with SRB

**DOI:** 10.1371/journal.pone.0261823

**Published:** 2022-01-19

**Authors:** Xuying Guo, Zhiyong Hu, Saiou Fu, Yanrong Dong, Guoliang Jiang, Ying Li

**Affiliations:** 1 College of Mining, Liaoning Technical University, Fuxin, China; 2 College of Science, Liaoning Technical University, Fuxin, China; 3 College of Civil Engineering, Liaoning Technical University, Fuxin, China; 4 Anshan Anqin Group, Anshan, China; National University of Singapore, SINGAPORE

## Abstract

The problems of acid mine drainage (AMD) in coal mine acidic wastewaters arise from a range of sources, including severe pollution with heavy metals and SO_4_^2-^ and difficulties during treatment. Based on the ability of Maifan stone to adsorb heavy metals and the dissimilatory reduction of SO_4_^2-^ by sulfate-reducing bacteria (SRB), Maifan stone-sulfate-reducing bacterium-immobilized particles were prepared via immobilization techniques using Shandong Maifan stone as the experimental material. The effects of Maifan stones containing SRB on mitigating AMD were investigated by constructing Dynamic Column 1 with Maifan stone-sulfate-reducing bacterium-immobilized particles and by constructing Dynamic Column 2 with SRB mixed with Maifan stones. By the use of adsorption isotherms, adsorption kinetics, a reduction kinetics model and X-ray diffraction (XRD) and scanning electron microscopy (SEM) studies, the mechanism by which Maifan stone-sulfate-reducing bacterium-immobilized particles mitigate AMD was revealed. The results showed that the total effect of Maifan stone-sulfate-reducing bacterium-immobilized particles on AMD was better than that of biological Maifan stone carriers. The highest rates for the removal of Fe^2+^, Mn^2+^, and SO_4_^2-^ in AMD were 90.51%, 85.75% and 93.61%, respectively, and the pH value of the wastewater increased from 4.08 to 7.64. The isotherms for the adsorption of Fe^2+^ and Mn^2+^ on Maifan stone-sulfate-reducing bacterium-immobilized particles conformed to the output of the Langmuir model. The adsorption kinetics were in accordance with Lagergren first-order kinetics, and the kinetics for the reduction of SO_4_^2-^ conformed to those of a first-order reaction model.

## 1. Introduction

Acid mine drainage (AMD) is polluted water produced by coal mining [1]. Derived from a wide range of sources, AMD exhibits complex water quality, low pH [2], large concentrations of SO_4_^2-^ and high concentrations of iron, manganese, copper, zinc and other heavy metals [3]. Direct discharge of AMD substantially pollutes water and soil resources, destroys the ecological environment, and threatens human health [4]. Currently, the methods commonly used for AMD treatment include the neutralization method [5], constructed wetland method [6,7], adsorption method [8] and microbial method. The adsorption method usually takes advantage of the characteristics of porosity and a large specific surface area of adsorbent to adsorb specific substances in solution to achieve purification [9]. E Wulandari et al. employed natural zeolite and synthetic zeolite to treat AMD, and the removal rates of Cu^2+^ reached 98.16% and 93.98%, respectively [10]. Zheng et al. used iron slag (FS) and carbon steel slag (CSS) from steel mills as adsorbents to remove sulfate from AMD; the results showed that the adsorption capacities of FS and CSS were 225.07 mg.g^-1^ and 320.57 mg.g^-1^, respectively [11]. The adsorption method has the advantages of simple operation, low cost and availability, but if the adsorption of heavy metal ions is not properly treated, secondary pollution is likely [12]. The microbial method mainly uses SRB to treat AMD [13,14], which can not only remove SO_4_^2-^ but also produce alkalinity to improve the pH of waste, which has the advantages of easy access and low treatment cost. Xiao Ye applied sulfate-reducing bacteria (SRB) to remove SO_4_^2-^ from acid mine wastewater and achieved a maximum removal rate of 80% [15]. Jennyfer used SRB to remove heavy metals such as As and Fe in AMD; the results showed that the removal rates of As and Fe were 73% and 78%, respectively, [16]. However, SRB had limited tolerance to high metal concentrations and low pH, and high acidity and high concentrations of heavy metal ions inhibit biological activity and harm biological organisms [17].

Microbial immobilization technology is a method that immobilizes microorganisms in a limited space by physical or chemical means to render them highly dense and maintain certain activity [18]. Microbial immobilization, polyvinyl alcohol (PVA), sodium alginate (SA), and other functional groups and carriers of the cementing material resulted in covalent bonds or van der Waals forces and other forms and main chain structure reinforcement. Microorganisms do not easily erode, ensuring that the high-density bacterial group within the microorganism-immobilized particles has high biological activity [18]. Currently, immobilized carrier materials for PVA and SA have large mechanical strength, good mass transfer performance and biological decomposition resistance and other properties. They also have good permeability, are nontoxic and have high transparency [19]. For microbial growth environments, a bacterial pollution system with adaptability and impact resistance ability is improved, and tolerance to heavy metals and pH is enhanced [20,21]. Hong used immobilized spheres to degrade chlorobenzene at a maximum rate of 78.16% [22]. Similarly, Mingliang Zhang [23] employed new immobilized sulfate-reducing bacterium beads that were prepared to treat AMD and obtained a maximum sulfate removal rate of 88%.

Maifan stone has good adsorption; is capable of bidirectional adjustment of the water pH, dissolution and biological activity; and has other advantages. Wen’s study showed that Maifan stone has a certain adsorption effect on Mn^2+^, Pb^2+^, Cd^2+^, Cr^3+^ and other metal ions [24], but the adsorption effect on SO_4_^2-^ is poor [25]. Zhang Lehong et al. showed that Maifan stone could dissolve a large number of beneficial trace elements, such as selenium and strontium; in particular, the increase rate of selenium reached 178.30%, and the increase rates of the major elements calcium and potassium were 30.30% and 912.50%, respectively [26]. Microorganisms can use these trace elements as nutritional factors to enhance their metabolic activity. Ma Puxi [27] determined that microorganisms can use trace elements released by Maifan stones as nutritional factors to enhance metabolic activity and the effect of denitrification. Jiang Honglin [28] used Maifan stone to prepare a moving bed biofilm reactor, which was applied in the in situ restoration of polluted rivers. The results showed that the chemical oxygen demand, ammonia nitrogen and total nitrogen removal rates of the moving bed biofilm reactor increased by 4.86%, 8.89% and 9.01%, respectively, after the addition of Maifan stone. Therefore, Maifan stone can be embedded during the preparation process of sulfate-reducing bacterium-immobilized particles to enhance the biological activity of SRB and improve the treatment effect of immobilized particles on AMD.

In this study, the adsorption method and microbial method were combined with SRB and Shandong Maifan stones using immobilization technology for combination and AMD treatment. The repair effect of Maifan stones and SRB on AMD was explored by constructing a Maifan stone- and sulfate-reducing bacterium-immobilized particle dynamic column and a Maifan stone-loaded sulfate-reducing bacterium dynamic column. The investigation can solve not only the technical bottleneck that single absorption metal ions of Maifan stone cannot remove sulfate root but also the inhibition of sulfate-reducing bacterial activity by heavy metal ions with low pH and high concentrations to ensure that the absorption of Maifan stone and sulfate-reducing bacterium biological activity are advantages of the process of treating acid coal mine wastewater. Combined with the absorption isotherm, adsorption kinetics, reduction kinetics model and X-ray diffraction (XRD), scanning electron microscopy (SEM) detection revealed the mechanism by which Maifan stone-sulfate-reducing bacterium-immobilized particles repair AMD.

## 2. Materials and methods

### 2.1 Experimental materials

Shandong Maifan stone: The main chemical constituents of Maifan stone from Linyi City, Shandong Province, China, are shown in Table 1. The Maifan stone was crushed and screened, and a sample with particle sizes ranging from 0.106–0.15 mm (100–150 mesh) was selected. The samples were washed three times with deionized water to remove impurities and then dried at 105°C.

**Table 1:** Main compositions of Shan Dong Maifan stone.

Constituent	SiO_2_	TiO_2_	Al_2_O_3_	Fe_2_O_3_	MnO	MgO	CaO	Na_2_O	K_2_O	P_2_O_5_
Maifan stone	67.90	0.32	15.75	2.82	0.06	0.66	2.51	5.45	1.59	0.056

SRB: Activated sludge from the Xihe River, Fuxin city, Liaoning Province, was selected as the strain screening sample. According to Liu’s method [29], the bacteria mixed with SRB as the dominant strain were enriched by modified Postgate B medium for subsequent experiments.

Corncob: In this experiment, corncobs was selected as the carbon source for immobilized particles. Corncob contains a rich mixture of organic components and mineral elements that can serve as nutrients for bacteria; it is a stable and inexpensive carbon source. Corncobs from Fuxin farmland with particle sizes ranging from 0.106–0.15 mm were selected.

AMD: Based on the measured water quality data of mine water in a coal mining area of Fuxin City, Liaoning Province, the pH of simulated acid mine wastewater was set to 4, and the concentrations of SO_4_^2-^, Fe^2+^, Mn^2+^, Mg^2+^ and Ca^2+^ were 834.5 mg.L^-1^, 14 mg.L^-1^, 6 mg.L^-1^, 50 mg.L^-1^, and 50 mg.L^-1^, respectively.

All the chemicals selected for the experiments were analytical reagent grade.

### 2.2 Experimental method

#### 2.2.1 Preparation of immobilized particles

Nine percent PVA and 0.5% SA were placed in a beaker and stirred until no bubbles existed. SA (0.5%) was added to the bacterial solution of SRB, which was stirred until sticky and set aside. Maifan stone and corn cobs were added to the gel, and the mixture was extracted by a syringe. The mixture was uniformly dripped into saturated boric acid solution (containing 2% CaCl_2_, pH 6.0). After addition, the immobilized beads were cross-linked by stirring with a magnetic stirrer for 4 h and then cured.

#### 2.2.2 Construction of the dynamic test device and experimental method

**Fig 1:**
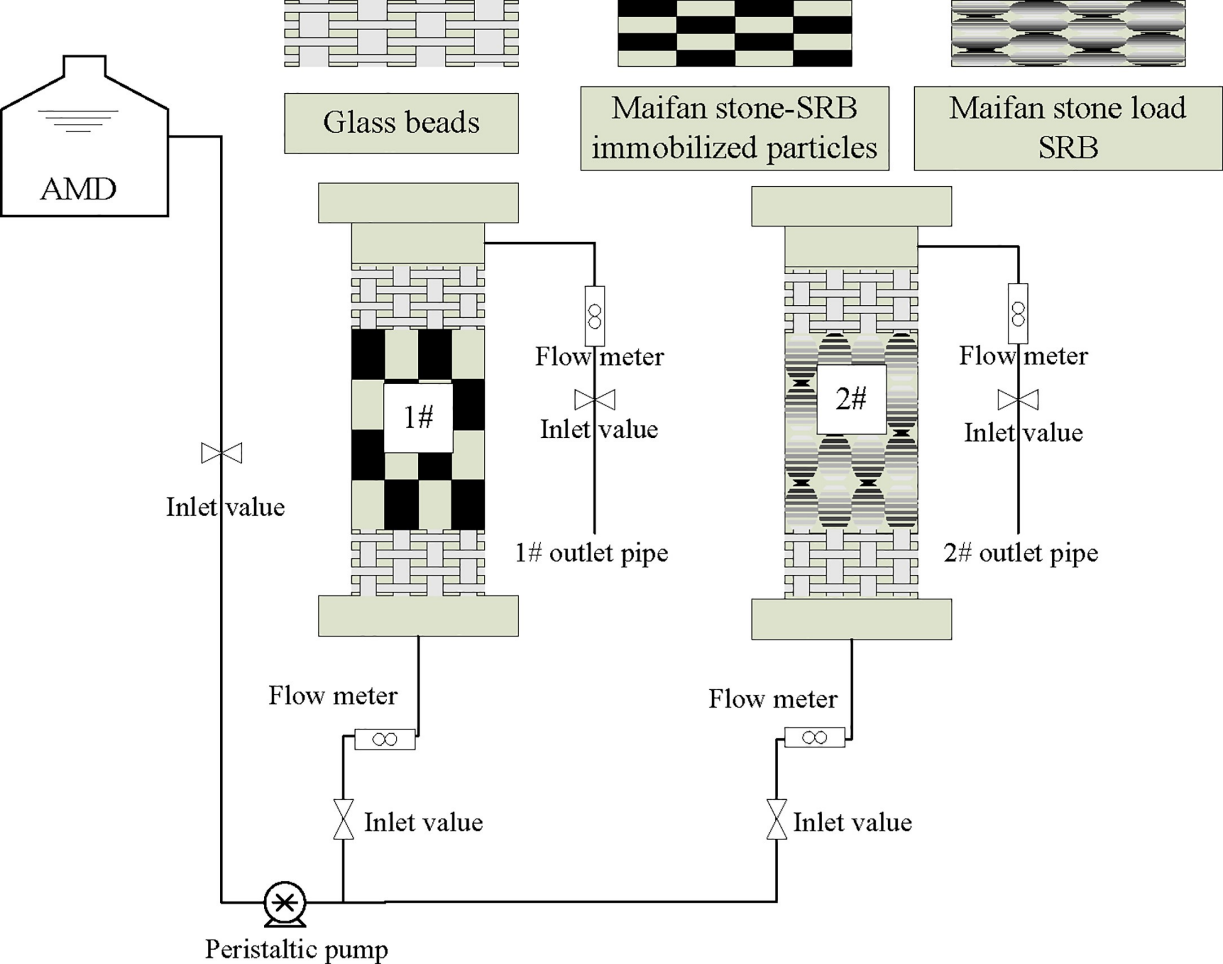
System diagram of the dynamic testing device.

A plexiglass tube with an inner diameter of 6 cm and a height of 50 cm was used to construct the dynamic column of Maifan stone-sulfate-reducing bacterium-immobilized particles and the dynamic column of SRB loaded by Maifan stone, as shown in Fig 1. Column 1 was filled with immobilized particles with a height of 40 cm, and Column 2 was filled with the same amount of Maifan stone, SRB and corn cob. Glass beads were added at the upper and lower ends of the dynamic columns and served as buffer protection layers. AMD was imported into the lower inlet of the dynamic column by a peristaltic pump; the hydraulic load in the dynamic column was controlled at 0.314 m^3^/(m^2^/day); and the hydraulic retention time was 12.21 h. Samples of water were collected regularly every day. The pH values and residual concentrations of Fe^2+^, Mn^2+^ and SO_4_^2-^ in the effluent were determined. Each group of experiments was repeated 3 times, and the mean value was obtained. The formula for calculation of removal efficiency η is expressed as follows:

$$\eta =\frac{C_{0}‐C_{t}}{C_{0}}\times 100\%$$ (1)

where C_0_ (mg.L^-1^) is the initial concentration of Fe^2+^, Mn^2+^ or SO_4_^2-^ and C_t_ (mg.L^-1^) is the residual concentration of Fe^2+^, Mn^2+^ or SO_4_^2-^ in the effluent of the dynamic column at time t.

#### 2.2.3 Test method for adsorption kinetics of Fe^2+^ and Mn^2+^

Solutions containing 30 mg.L^-1^ Fe^2+^ and 15 mg.L^-1^ Mn^2+^ were prepared. Next, 100 mL of solution was added to a 250 mL conical flask, and the immobilized particles were added at a ratio of 1:10 (g.mL^-1^). After shaking for 1, 2, 3, 4, and 5 days at 35°C and 150 r/min, the residual concentrations of Fe^2+^ and Mn^2+^ in the solution were determined, and the adsorption capacity *q* of Maifan stone-sulfate-reducing bacterium-immobilized particles for Fe^2+^ and Mn^2+^ was calculated. Each group of experiments was repeated 3 times, and the mean value was obtained.

#### 2.2.4 Adsorption isotherms of Fe^2+^and Mn^2+^

Fe^2+^ isothermal adsorption test: 100 mL of solutions with different Fe^2+^ concentrations (initial concentrations were 10 mg.L^-1^, 20 mg.L^-1^, 30 mg.L^-1^, 40 mg.L^-1^ and 50 mg.L^-1^) were added to 250 mL conical flasks, and immobilized particles were added to give solid-liquid ratios of 1:10 (g/mL). After oscillation at 35°C and 150 r/min for 5 days, the concentration of Fe^2+^ remaining in the solution was determined, and the adsorption capacity *q* of Maifan stone-sulfate-reducing bacterium-immobilized particles for Fe^2+^ was calculated. Each group of experiments was repeated 3 times, and the mean value was obtained.

Isothermal adsorption of Mn^2+^: Preparation of different concentrations of Mn^2+^ solution (initial concentrations were 5 mg.L^-1^, 10 mg.L^-1^, 15 mg.L^-1^, 20 mg.L^-1^, 25 mg.L^-1^); the other conditions were the same as those previously described. The formula for calculation of adsorption *q* was

$$q=\frac{(C_{0}‐C_{t})\times V}{m}$$ (2)

where C_0_ is the initial concentration of ions to be measured (mg.L^-1^), C_t_ is the residual concentration after adsorption (mg.L^-1^), V is the solution volume (L), and m is the mass of immobilized particles (g).

#### 2.2.5 Water quality detection and material characterization methods

Fe^2+^ concentrations were determined by o-phenanthroline spectrophotometry (HJ/T 345–2007), Mn^2+^ content was determined by potassium periodate spectrophotometry (GB 11906–89), SO_4_^2-^ content was determined by barium chromate spectrophotometry (HJ/T 342–2007), pH was determined with the glass electrode method (GB 6920–86), and Eh was measured by a Pen redox potentiometer.

SEM (3400 N) was employed to analyze the morphological properties of Shandong Maifan stones, Maifan stone-sulfate-reducing bacterium-immobilized particles, Maifan stone-sulfate-reducing bacterium-immobilized particles after AMD treatment and Maifan stone-loaded SRB after AMD treatment. XRD (D/MAX2400) was used to analyze the mineral compositions of Shandong Maifan stones, Maifan stone-sulfate-reducing bacterium-immobilized particles, Maifan stone-sulfate-reducing bacterium-immobilized particles after AMD treatment, and Maifan stone-loaded SRB after AMD treatment.

## 3. Results and discussion

### 3.1 Dynamic test of Maifan stones combined with SRB for mitigation of AMD

**Fig 2:**
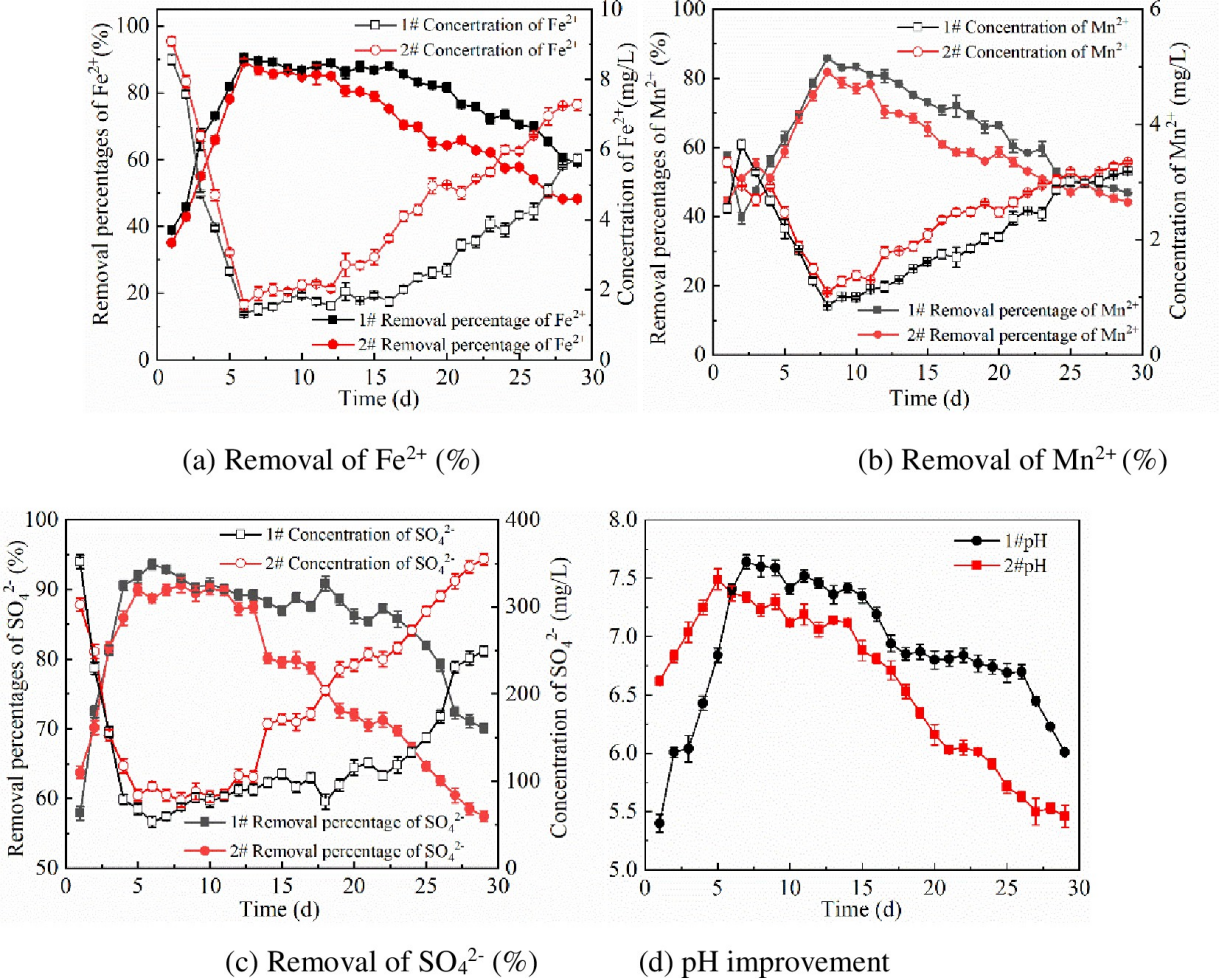
Removal effect of Dynamic Columns 1 and 2 with AMD. (a) Removal of Fe^2+^ (%). (b) Removal of Mn^2+^ (%). (c) Removal of SO_4_^2-^ (%). (d) pH improvement.

The experimental results for the column containing sulfate-reducing bacterium-immobilized particles (Dynamic Column 1) and the column containing Maifan stone loaded with SRB (Dynamic Column 2) in treating AMD are shown in Fig 2.

#### 3.1.1 Removal percentage of Fe^2+^

Fig 2(a) shows that the percentages of Fe^2+^ in AMD removed by Dynamic Columns 1 and 2 initially increased and then decreased. From 1 to 6 days, the percentages of Fe^2+^ removal by Dynamic Column 1 and Dynamic Column 2 increased from 38.79% and 35.04% to 90.51% and 88.73%, respectively. The removal of Fe^2+^ mainly depends on the adsorption of Maifan stone [30], ion exchange [31] and precipitation. SRB produces S^2−^ [32] due to the reduction [33] of SO_4_^2−^, which forms a precipitate with Fe^2+^. Additionally, the removal of Fe^2+^ was enhanced because the negative charge on the surface of SRB [34] facilitates electrostatic adsorption of Fe^2+,^ and the sulfate-reducing bacterium extracellular polymer also affects biological flocculation of Fe^2+^ [35]. The removal of Fe^2+^ by Dynamic Column 1 stabilized in 7–15 days; and the removal of Fe^2+^ by Dynamic Column 2 continued to evolve. The removal of Fe^2+^ in AMD with Dynamic Column 1 was more effective than that with Dynamic Column 2, which indicated that the technology based on immobilized particles prolonged the biological activity of SRB and improved the removal efficiency of Fe^2+^.

During the period 20–30 days, the levels of Fe^2+^ removal by Columns 1 and 2 decreased gradually because the pores of Maifan stones were blocked after substantial periods of adsorption, thus affecting adsorption. The gradual accumulation of heavy metals in wastewater would make the wastewater toxic toward SRB and serve to reduce sulfate-reducing bacterial activity, thereby inhibiting the removal of Fe^2+^.

#### 3.1.2 Removal percentage of Mn^2+^

Fig 2(b) shows that the efficiency of Mn^2+^ removal from AMD by Dynamic Columns 1 and 2 initially increased and then decreased. From day 1 to day 8, the removal efficiencies increased from 57.53% and 44.28% to 85.75% and 81.83%, respectively. On the 8th day, the residual Mn^2+^ concentrations were 0.86 mg.L^-1^ and 1.09 mg.L^-1^, respectively. On the 8th day, the removal efficiency for Mn^2+^ began to decrease. The efficiency of Mn^2+^ removal was lower than that of Fe^2+^ because the mechanisms for adsorption of Fe^2+^ and Mn^2+^ in the system differed [36]. SRB in columns 1 and 2 reduced SO_4_^2-^ to S^2-^, which combined with Mn^2+^ to form MnS precipitates. When Mn^2+^ in the system accumulated to a certain concentration, SRB began to die, thereby affecting the removal of Mn^2+^. In addition, due to its large solubility constant, MnS easily dissolves in a weakly acidic environment, and Mn^2+^ cannot be completely removed by precipitation. Therefore, the efficiency of Mn^2+^ removal is lower than that of Fe^2+^.

#### 3.1.3 Removal percentage of SO_4_^2-^

Fig 2(c) shows that the removal of SO_4_^2-^ in AMD by Dynamic Columns 1 and 2 initially increased and then decreased, and column 1 was significantly better than column 2. From 1 to 6 days, the SO_4_^2-^ removal efficiency of Dynamic Column 1 increased from 57.86% to 93.61%. On days 1–8, the removal efficiency of Dynamic Column 2 increased from 63.74% to 90.62%. Since the corncobs in the system released organic matter with nutrients supporting the growth of SRB, a sufficient carbon source and appropriate COD/SO_4_^2-^ enhanced the activity of SRB in the system [37]. This finding is conducive to the dissociation and reduction of SO_4_^2-^ and thereby enhances the removal of SO_4_^2-^, which shows an upward trend. On days 7–22, the performance of Dynamic Column 1 tended to stabilize.

On days 9–13, dynamic column 2 tended to stabilize. The efficiency of column 1 during the period of stability was better than that of column 2, which indicated that immobilization technology slowly released organic matter and improved sulfate-reducing bacterium biological activity over the long term. At later stages of the reaction, the removal efficiency of SO_4_^2−^ decreased due to a gradual decrease in the amount of organic matter released and the inhibition of sulfate-reducing bacterial activity caused by high concentrations of heavy metal ions resulting from continuous inflow. Comprehensive analysis of SO_4_^2-^ removal indicated that the efficiency of column 1 was greater than that of column 2.

#### 3.1.4 pH improvement

Fig 2(d) shows that Dynamic Columns 1 and 2 initially affected increases and then affected decreases in the pH of AMD. Columns 1 and 2 increased the initial pH value from 4.08 to 7.64 and 7.49, respectively. At the early stages, the main factor causing increases in pH values was the ability of Maifan stones to affect bidirectional regulation [38]. From 1 to 5 days, the pH of the AMD treated by Dynamic Column 2 increased more than that of AMD treated in Dynamic Column 1, because direct treatment of wastewater by SRB consumed H^+^ [39] and the pH of the wastewater increased significantly. Simultaneously, SRB decomposes the carbon source in the environment to produce HCO^3-^ through biological metabolism, which increases the pH value and alkalinity of the solution [39]. After 5 days, the HCO^3-^ content increases with a continuous anaerobic reaction of sulfate-reducing bacterium-dominated sulfate reduction [40]. The effluent pH of Dynamic Column 1 continued to rise, stabilized and then decreased slowly; the pH of the effluent treated in Dynamic Column 2 continued to decline. The improvement in pH affected by Dynamic Column 1 was significantly better than that of Dynamic Column 2.

#### 3.1.5 Effect analysis of repairing AMD

A comprehensive analysis showed that during the use of Dynamic Columns 1 and 2 for the removal of Fe^2+^, Mn^2+^, and SO_4_^2-^ and an increase in pH, the performance of Column 1 was better than that of Column 2. The highest removal rates of Fe^2+^, Mn^2+^ and SO_4_^2-^ by dynamic column 1 were 90.51%, 85.75% and 93.61%, respectively, and the pH value of wastewater increased from 4.08 to 7.64. Previous studies showed that when Maifan stone is used to treat AMD, the removal rates of SO_4_^2-^, Fe^2+^ and Mn^2+^ in mine acid wastewater are 6.87%, 68.9% and 32.8%, respectively [41]. The average removal rates of SO_4_^2-^ and Mn^2+^ by SRB for AMD were 61.63% and 72.35%, respectively [42]. The effect of direct remediation of Fe^2+^, Mn^2+^ and SO_4_^2-^ pollutants in AMD with Dynamic Column 1 and Maifan stone was analyzed. The results confirmed that immobilization technology could improve the adaptability, shock resistance and low pH tolerance of the bacteria in the polluted system to improve the remediation effect.

### 3.2 Adsorption isotherms and kinetic analysis of immobilized particles

#### 3.2.1 Analysis of adsorption isotherms for Fe^2+^ and Mn^2+^

**Fig 3:**
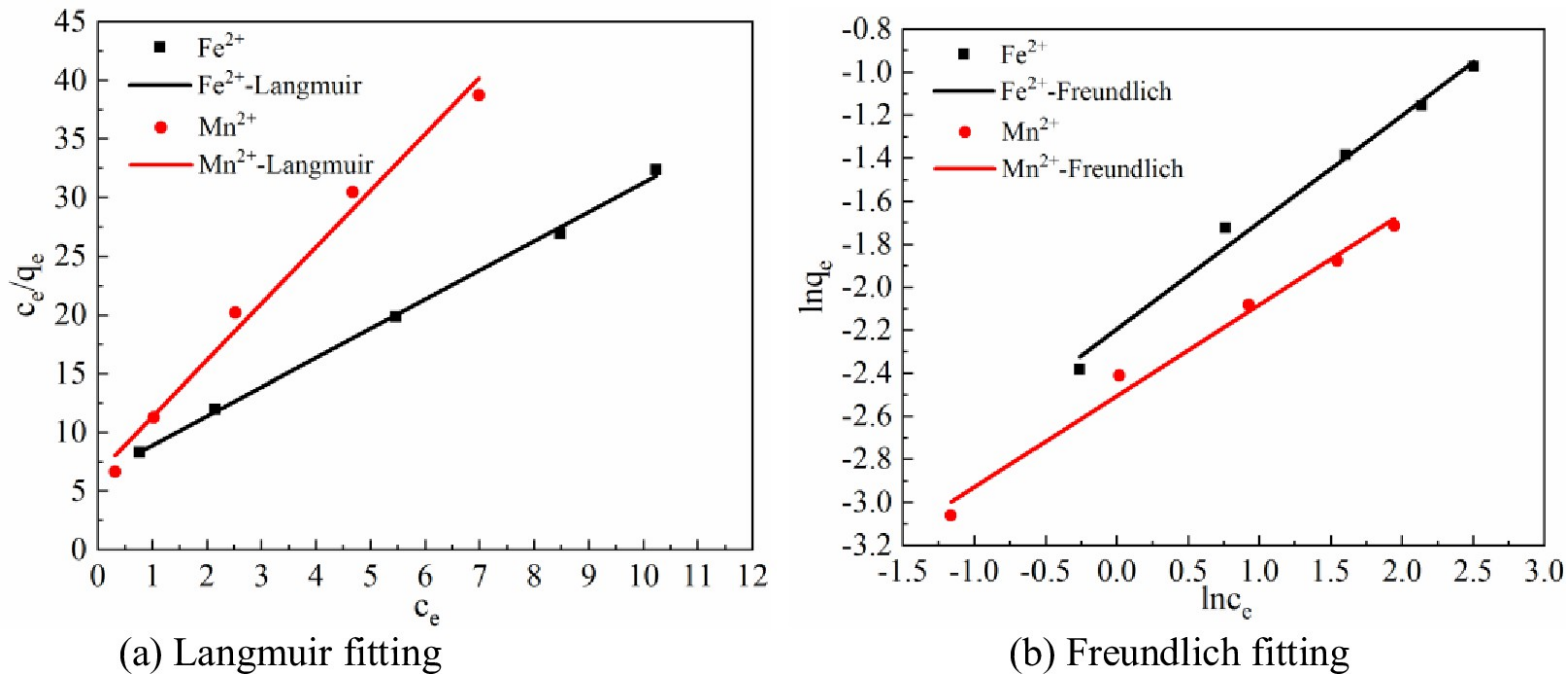
Isotherms for adsorption of Fe^2+^ and Mn^2+^ on immobilized particles. (a) Langmuir fitting. (b) Freundlich fitting.

Langmuir and Freundlich adsorption isotherm models were used to analyze the adsorption isotherms of Fe^2+^ and Mn^2+^ on Maifan stone-sulfate-reducing bacterium-immobilized particles. The fitting results are shown in Fig 3.

$$Langmuir\;adsorption\;isotherm\;equation:\;\frac{c_{e}}{q_{e}}=\frac{1}{q_{m}k_{L}}+\frac{c_{e}}{q_{m}}$$ (3)

where q_e_ (mg.g^-1^) is the amount of metal ions adsorbed per unit mass of adsorbent at equilibrium; c_e_(mg.L^-1^) is the equilibrium concentration of solute in the bulk solution; q_m_ is the saturated adsorption capacity; and *K*_*L*_ is the Langmuir model adsorption constant.

$$\mathrm{Freundlich}\;\mathrm{adsorption}\;\mathrm{isotherm}\;\mathrm{equation}:\;q_{e}=k_{f}c_{e}{}^{1/n}$$ (4)

where q_e_ (mg.g^-1^) is the amount of metal ions adsorbed per unit mass of adsorbent at equilibrium, Ce (mg.L^-1^) is the equilibrium concentration of solute in the bulk solution, *k*_*L*_ is the Freundlich model adsorption constant, and n is the correlation coefficient of adsorption strength.

According to Fig 3, the Langmuir fit to the curve for the removal of Fe^2+^ by Maifan stone-sulfate-reducing bacterium-immobilized particles was c_e_/q_e_ = 6.43737c_e_+2.48397, and R^2^ = 0.99754. The Freundlich fitting curve equation was lnq_e_ = 0.49634lnce-2.19336, R^2^ = 0.9859. The Langmuir fitting curve equation for Mn^2+^ removal by the Maifan stone-sulfate-reducing bacterium-immobilized particles was c_e_/q = 4.8172c_e_+6.54707, R^2^ = 0.98428. The Freundlich fitting curve equation was lnq_e_ = 0.42396lnc_e_-2.50475, R^2^ = 0.98332. A comparison of the correlation coefficients R^2^ reveal that those for the Langmuir fits for adsorption of Fe^2+^ and Mn^2+^ by the immobilized particles were higher, so the adsorption of Fe^2+^ and Mn^2+^ in solution by the immobilized particles was more consistent with the Langmuir model. This finding indicated that the adsorption process of Fe^2+^ and Mn^2+^ by particles was dominated by monolayer adsorption. As the adsorption sites were gradually occupied, the adsorption rate gradually decreased until equilibrium was reached.

Comparing the saturated adsorption capacity *q*_*m*_, the adsorption capacity of Maifan stone-sulfate-reducing bacterium-immobilized particles on Fe^2+^ is stronger. The Freundlich constants of Fe^2+^ and Mn^2+^ are 0.49634 and 0.42396, respectively; both are less than 0.5. This finding shows that the adsorption of Fe^2+^ and Mn^2+^ is relatively easy for Maifan stone-sulfate-reducing bacterium-immobilized particles.

#### 3.2.2 Analysis of adsorption kinetics for Fe^2+^ and Mn^2+^

**Fig 4:**
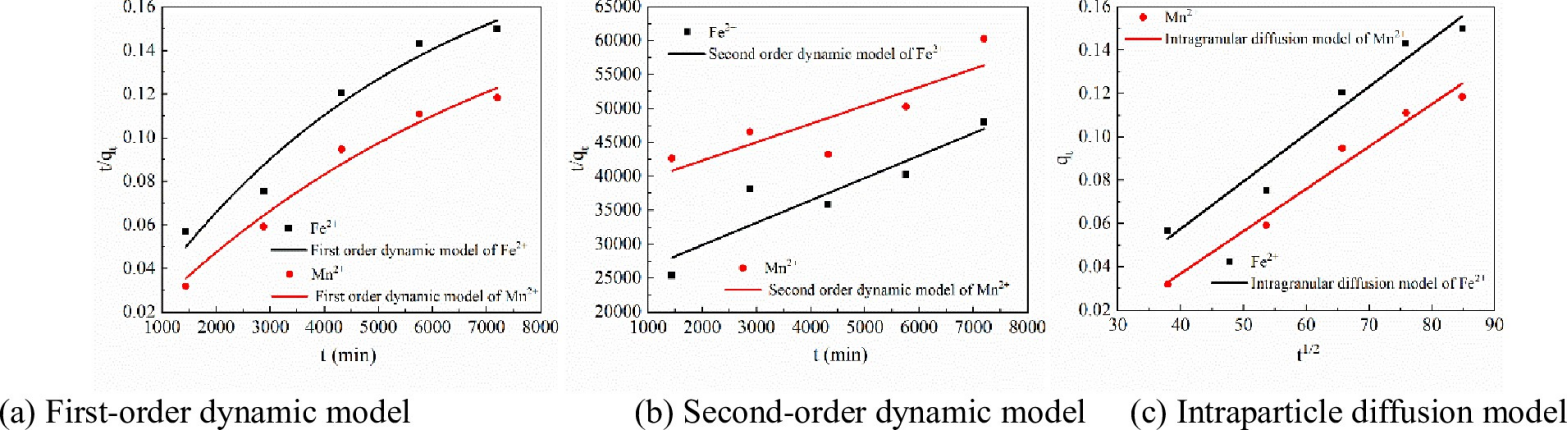
Results for fitting kinetics for adsorption of Fe^2+^ and Mn^2+^ on immobilized particles. (a) First-order dynamic model. (b) Second-order dynamic model. (c) Intraparticle diffusion model.

The Lagergren first-order dynamic model, Lagergren second-order dynamic model and intraparticle diffusion model were used to analyze the kinetics for the adsorption of Fe^2+^ and Mn^2+^ on Maifan stone-sulfate-reducing bacterium-immobilized particles. The fitting results are shown in Fig 4 and Table 2.

$$First‐order\;model:\;ln(q_{e}‐q_{t})=\ln q_{e}‐k_{1}t$$ (5)

where q_e_ (mg.g^-1^) and qt (mg.g^-1^) are the amounts of adsorbed adsorbate at equilibrium and at time t, respectively, and k1 (min^-1^) is the rate constant of pseudo-first-order adsorption.

**Table 2:** Kinetic parameters of adsorption of Fe^2+^ and Mn^2+^ by immobilized particles.

kinetic model	parameter	Fe^2+^	Mn^2+^
First-order dynamic model	*k*_1_	1.94074×10^−4^	1.41278×10^−4^
	*q*_*e*_	0.20428	0.1924
	*R* ^2^	0.94912	0.97054
Second-order dynamic model	*k*_2_	4.66513×10^−4^	1.44388×10^−4^
	*q*_*e*_	0.30337	0.41585
	*R* ^2^	0.78206	0.64092
Intraparticle diffusion model	*k*_*p*_	0.00219	0.00196
	*c*	-0.03005	-0.0414
	*R* ^2^	0.94433	0.9678

$$Second‐order\;model:\;\frac{t}{q_{t}}=\frac{1}{k_{2}q_{e}{}^{2}}+\frac{t}{q_{e}}$$ (6)

where *k*_2_ (mg.(g.min))^-1^ is the equilibrium rate constant of pseudo-second-order adsorption and qe (mg. g^-1^) and qt (mg.g^-1^) are the amounts of adsorbed adsorbate at equilibrium and at time t.

$$\mathrm{Intraparticle}\;\mathrm{diffusion}\;\mathrm{model}:\;q_{t}=k_{p}t^{1/2}$$ (7)

where q_t_ (mg.g^-1^) is the amount of solute on the surface of the sorbent at time t and k_p_ (mg.(g.min^1/2^)^-1^) is the intraparticle diffusion rate constant.

Table 2 and Fig 4 show that when the data for Fe^2+^ adsorption by Maifan stone-sulfate-reducing bacterium-immobilized particles were treated with the first-order adsorption kinetics model, second-order adsorption kinetics model and intraparticle diffusion model, the fit of the data yielded correlation coefficients R^2^ of 0.94912, 0.78206 and 0.94433, respectively. The first-order kinetic model had a greater correlation coefficient, so the adsorption of Fe^2+^ by Maifan stone-sulfate-reducing bacterium-immobilized particles was more consistent with the first-order kinetic model: q_t_ = *q*_*t*_ = 0.20428×(1-e^-1.94074×10-4*t*^), R^2^ = 0.98428. When Mn^2+^ was adsorbed by Maifan stone-sulfate-reducing bacterium-immobilized particles, the first-order adsorption kinetic model (R^2^ = 0.97054) exhibited a greater correlation coefficient than the second-order adsorption kinetic model (R^2^ = 0.64092) and intraparticle diffusion model (R^2^ = 0.9678). Therefore, the adsorption of Mn^2+^ by Maifan stone-sulfate-reducing bacterium-immobilized particles was also more consistent with the first-order adsorption kinetics model: *q*_*t*_ = 0.1924×(1-e^-1.41278×10-4*t*^), R^2^ = 0.97054. The *q*_*e*_ obtained by fitting the equations was greater than the amount of equilibrium adsorption at the experimental node, that is, at t = 5 days, indicating that the adsorption of Fe^2+^ and Mn^2+^ by immobilized particles did not reach the saturation state.

#### 3.2.3 Analysis of reduction kinetics test results of SO_4_^2-^

**Fig 5:**
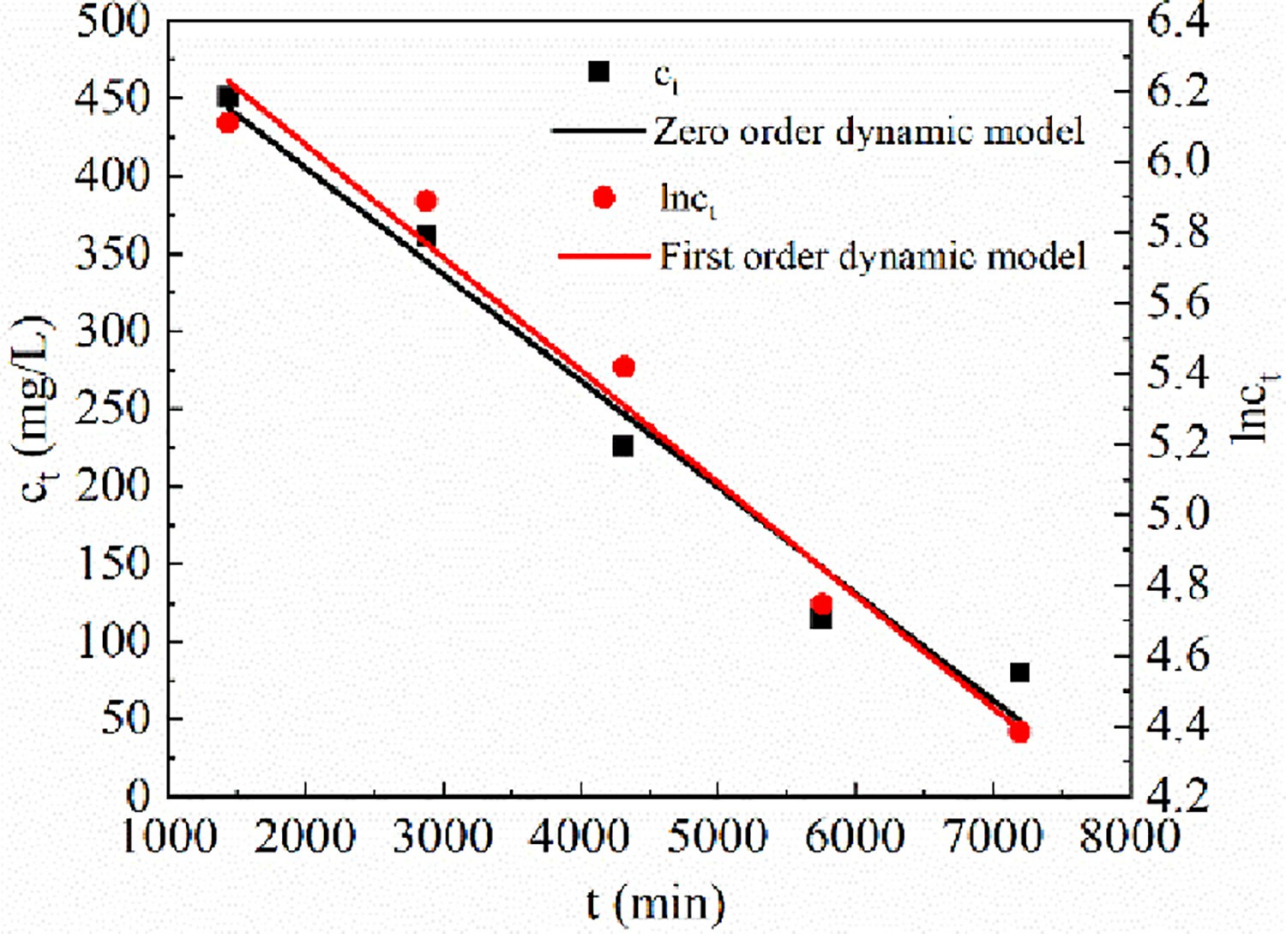
First-order kinetic and zero-order kinetic models of SO_4_^2-^.

First-order kinetic and zero-order kinetic models were employed to analyze the reduction of SO_4_^2-^ by Maifan stone-sulfate-reducing bacterium-immobilized particles. The fitting results are shown in Fig 5.

Fig 5 shows that the equation for the zero-order dynamic fit to data from the reduction of SO_4_^2-^ by immobilized particles was *c*_*t*_ = 542.828–0.06856 t, R^2^ = 0.96314, and the first-order dynamic fitting equation was ln*c*_*t*_ = 6.68937–3.19398×10^−^*4 t*, R^2^ = 0.96865. The correlation coefficient for the first-order kinetics fit was greater than that of the zero-order kinetics fit, indicating that the reduction of SO_4_^2-^ by immobilized particles is more consistent with the first-order kinetics model. Furthermore, the electron acceptor was the main factor affecting the reduction of SO_4_^2-^ by SRB.

### 3.3 Mechanistic analysis of AMD mitigation by Maifan stones combined with SRB

#### 3.3.1 XRD analysis

**Fig 6:**
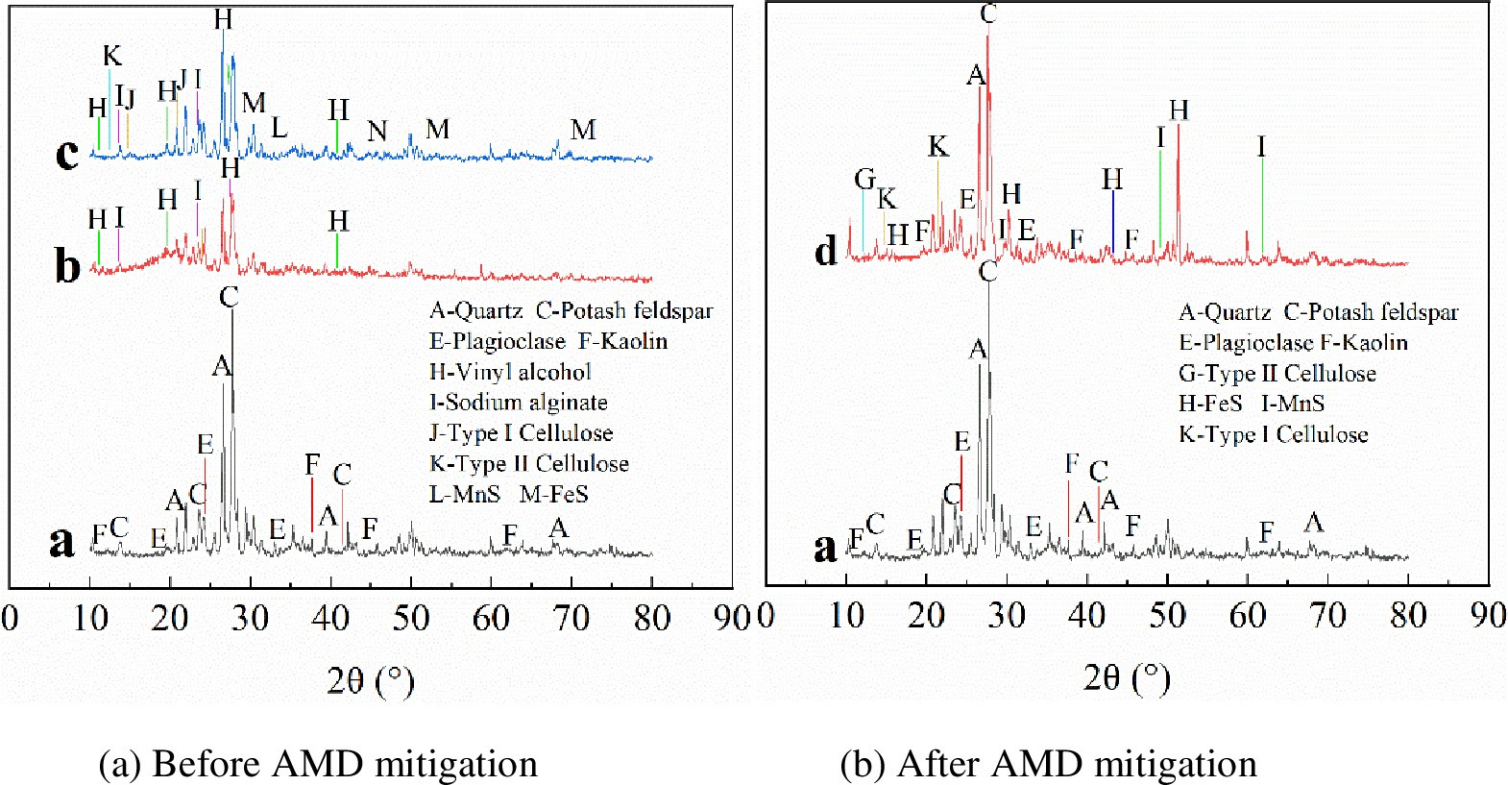
XRD patterns of Maifan stones combined with SRB before and after AMD repair. (a) Before AMD mitigation. (b) After AMD mitigation. a: Shandong Maifan stone; b: Before treatment of AMD with Maifan stone sulfate-reducing bacterium-immobilized particles; c: After treatment of AMD with Maifan stone sulfate-reducing bacterium-immobilized particles; d: Treatment of AMD by SRB loaded with Maifan stone.

Fig 6 shows the XRD data for Shandong Maifan stones combined with Maifan stone-sulfate-reducing bacterium-immobilized particles and with SRB loaded with Maifan stones, before and after AMD mitigation.

Fig 6(a) shows that Maifan stone-sulfate-reducing bacterium immobilized particles exhibit typical peak characteristics of Maifan stones, such as those for quartz and potash feldspar, plagioclase, kaolin, PVA and SA [43]. The immobilized particles of Maifan stone-sulfate-reducing bacterium and Maifan stone showed similar XRD patterns, indicating that the immobilization technology had not changed the original crystal structure of Maifan stone. Fig 6(b) shows diffraction peaks for quartz and potash feldspar, plagioclase, kaolin, type I cellulose and type II cellulose that appeared in the XRD spectra of AMD treated by Maifan stone loaded with SRB. Typical peaks characteristic of type I cellulose and type II cellulose appeared after AMD was treated with immobilized particles [44,45], indicating that the corncobs carbon source slowly released organic matter for the growth and metabolism of SRB. However, MnS and FeS peaks were observed after AMD treatment, indicating that SRB used corncobs as a carbon source to metabolize and reduce SO_4_^2-^ to S^2^, which reacted with heavy metal ions in AMD to form sulfide precipitates.

#### 3.3.2 SEM analysis

**Fig 7:**
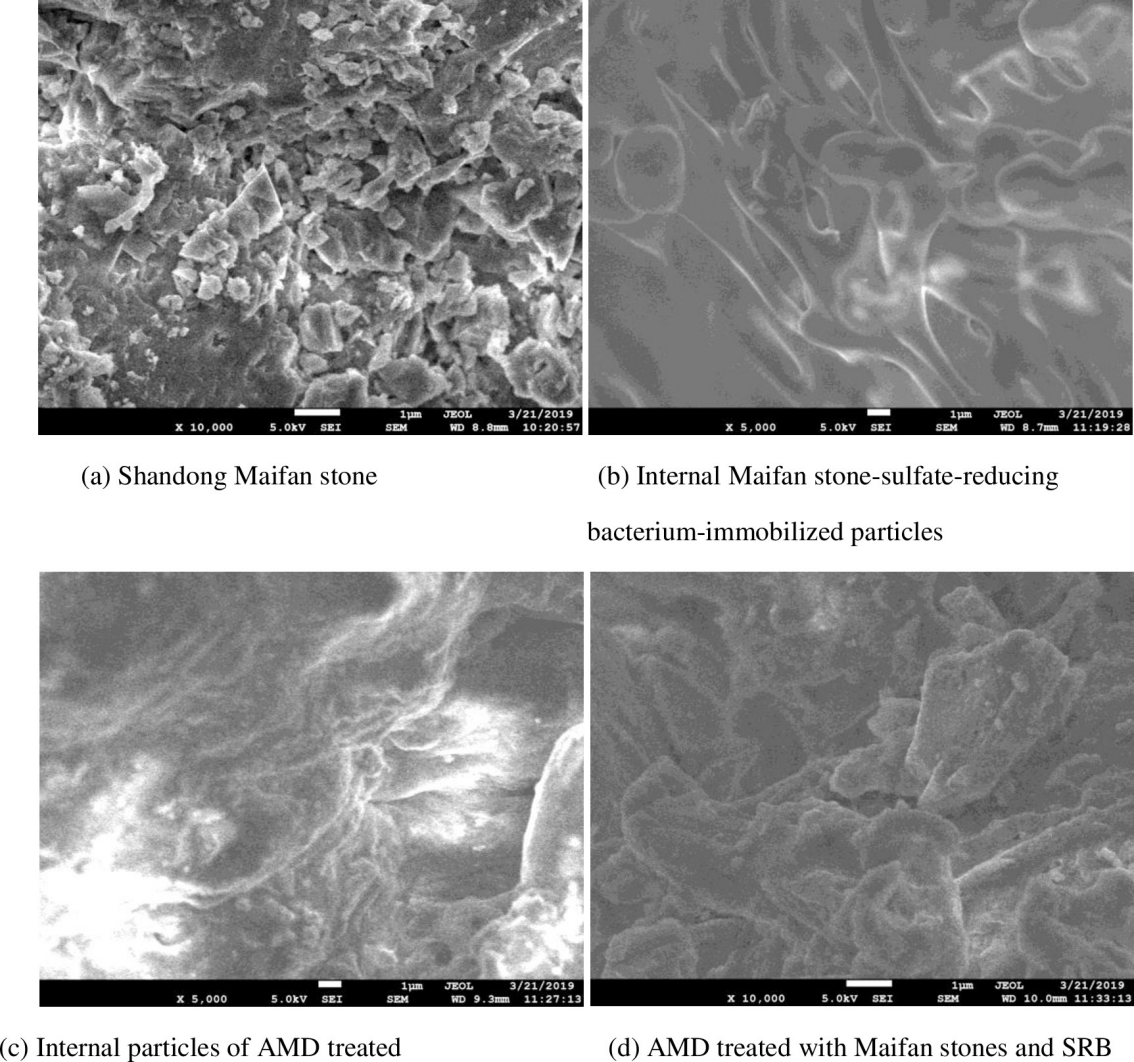
SEM images of Maifan stones combined with SRB before and after AMD repair. (a) Shandong Maifan stone. (b) Internal Maifan stone-sulfate-reducing bacterium-immobilized particles. (c) Internal particles of AMD treated. (d) AMD treated with Maifan stones and SRB.

The interior of Shandong Maifan stone and Maifan stone-sulfate-reducing bacterium-immobilized particles before and after AMD treatment and a scanning electron micrograph diagram of Maifan stone combined with SRB for the treatment of AMD are shown in Fig 7.

Fig 7(b) shows that the internal texture of Maifan stone-sulfate-reducing bacterium-immobilized particles is uniform and that pores developed, indicating that they had strong biological activity and basically met the requirements for AMD treatment. A comparison of Fig 7(b) and 7(c) shows that the internal structure of Maifan stone-sulfate-reducing bacterium-immobilized particles before and after the reaction underwent a great change. After the reaction part of the surface of the pore decreases in size and becomes uneven, the internal impurities increase, and the pores decrease. This finding shows that the removal of SO_4_^2-^ by the Maifan stone-sulfate-reducing bacterium system occurs simultaneously on the surface and inside the particles. SO_4_^2-^ enters the immobilized particles from the external water environment through the pores and forms S^2-^ by the sulfate-reducing bacterial dissimilatory reduction reaction. Fe^2+^ and Mn^2+^ in AMD combine with S^2-^ and produce sulfide precipitation in the particles, causing an increase in internal impurities. The pores decrease and even clogged. Many raised folds simultaneously formed. This phenomenon is caused by the toxic effect of Mn^2+^ adsorbed on the surface on SRB, which reduces the biological activity of SRB and produces a surface folding morphology [46]. In the Maifan stone-sulfate-reducing bacterium immobilized system, a series of physical, chemical and biological reactions occurred in the processing of AMD. Thus, the pollution of ions in the form of precipitation in the internal pores effectively inhibits the diffusion of pollutants. A comparison of Fig 7(a) and 7(d) reveals that after the dynamic test treatment of Maifan stone particles in AMD, which involves prolonged exposure to an acidic water environment their structure is eroded and destroyed and becomes looser, with a large amount of particle material on the surface. The results show that the sulfate-reducing bacterial dynamic column is not suitable for long-term reaction processes. A comprehensive comparison shows that Maifan stone sulfate-reducing bacterium-immobilized particles are more conducive to long-term resistance to AMD pollution load.

## 4. Conclusions

In this study, using the adsorption of Maifan stone and the reduction characteristics of SRB, based on immobilization technology, Maifan stone-sulfate-reducing bacterium-immobilized particles were employed to repair the pollution of Fe^2+^, Mn^2+^, and SO_4_^2-^ in the acidic wastewater of coal mines. This approach can not only improve the activity of SRB to remove heavy metal cations and sulfate anions in AMD but also compensate for the lack of a single adsorption cation of Maifan stone. The synergistic effect of SRB and Maifan stones is more effective with a low cost. Based on the dynamic test and principles of dynamics and thermodynamics, the adsorption kinetics and adsorption thermodynamics equations of the treatment of AMD with Maifan stone and sulfate-reducing bacterium-immobilized particles were proposed for the first time, revealing the mechanism of the efficient repair of AMD with Maifan stone and sulfate-reducing bacterium-immobilized particles. The following conclusions are presented:

1. By constructing Maifan stone-sulfate-reducing bacterium-immobilized particles in Dynamic Column 1 and Maifan stone-loaded SRB in Dynamic Column 2, the effects of Maifan stone-sulfate-reducing bacterium-immobilized particles and Maifan stone-loaded SRB on the remediation of Fe^2+^, Mn^2+^ and SO_4_^2-^ in AMD were compared. The Maifan stone-sulfate-reducing bacterium-immobilized particles in Dynamic Column 1 exhibited a better treatment effect, and the efficiencies for the removal of Fe^2+^, Mn^2+^ and SO_4_^2-^ were 90.51%, 85.75% and 93.61%, respectively. The pH of the wastewater was increased from 4.08 to 7.64.
2. The adsorption of Fe^2+^ and Mn^2+^ by Maifan stone-sulfate-reducing bacterium-immobilized particles conformed to the Langmuir adsorption isotherm model and first-order adsorption kinetics model. The Langmuir curve fitting equation for Fe^2+^ was c_e_/q_e_ = 6.43737c_e_+2.48397 and R^2^ = 0.99754. The Langmuir curve fitting equation for Mn^2+^ was c_e_/q_*e*_ = 4.8172c_e_+6.54707, and R^2^ = 0.98428. The equation for the first-order fit to the data for adsorption of Fe^2+^ was *q*_*t*_ = 0.20428×(1-e^-1.94074×10-4*t*^), and R^2^ = 0.98428. The equation for the first-order fit to the data for adsorption of Mn^2+^ was *q*_*t*_ = 0.1924×(1-e^-1.41278×10-4*t*^), and R^2^ = 0.97054.
3. The process of SO_4_^2-^ reduction by SRB exhibited first-order reaction kinetics, and the fitting equation was ln*c*_*t*_ = 6.68937–3.19398×10^−4^ t, with R^2^ = 0.96865, indicating that the electron acceptor was the main factor affecting the reduction of SO_4_^2-^ by SRB.

## Supporting information

S1 Data (XLSX)
